# Single Cell Label-Free Probing of Chromatin Dynamics During B Lymphocyte Maturation

**DOI:** 10.3389/fcell.2021.646616

**Published:** 2021-03-26

**Authors:** Rikke Morrish, Kevin Ho Wai Yim, Stefano Pagliara, Francesca Palombo, Richard Chahwan, Nicholas Stone

**Affiliations:** ^1^School of Physics and Astronomy, University of Exeter, Exeter, United Kingdom; ^2^Living Systems Institute and School of Biosciences, University of Exeter, Exeter, United Kingdom; ^3^Institute of Experimental Immunology, University of Zurich, Zurich, Switzerland

**Keywords:** B cell, auxeticity, nuclear architecture, chromatin, vibrational spectroscopy, microfluidics

## Abstract

Large-scale intracellular signaling during developmental growth or in response to environmental alterations are largely orchestrated by chromatin within the cell nuclei. Chemical and conformational modifications of the chromatin architecture are critical steps in the regulation of differential gene expression and ultimately cell fate determination. Therefore, establishing chemical properties of the nucleus could provide key markers for phenotypic characterization of cellular processes on a scale of individual cells. Raman microscopy is a sensitive technique that is capable of probing single cell chemical composition—and sub-cellular regions—in a label-free optical manner. As such, it has great potential in both clinical and basic research. However, perceived limitations of Raman spectroscopy such as low signal intensity and the difficulty in linking alterations in vibrational signals directly with ensuing biological effects have hampered advances in the field. Here we use immune B lymphocyte development as a model to assess chromatin and transcriptional changes using confocal Raman microscopy in combination with microfluidic devices and correlative transcriptomics, thereby linking changes in chemical and structural properties to biological outcomes. Live B lymphocytes were assessed before and after maturation. Multivariate analysis was applied to distinguish cellular components within each cell. The spectral differences between non-activated and activated B lymphocytes were then identified, and their correlation with known intracellular biological changes were assessed in comparison to conventional RNA-seq analysis. Our data shows that spectral analysis provides a powerful tool to study gene activation that can complement conventional molecular biology techniques and opens the way for mapping the dynamics in the biochemical makeup of individual cells.

## Introduction

The ability to measure and quantify molecular changes during cellular development can enable the characterization of cells during differentiation, cellular responses to extracellular cues, or disease progression. Conventional techniques, such as fluorescent tagging of molecules visualized with fluorescence microscopy (Sanderson et al., 2014) and transcriptomics and proteomics profiling (Levy and Myers, 2016), have been extensively used to assess molecular changes occurring within cells. However, their stark limitations are the need for target labeling and/or destruction of the biological specimens under study. That is why, non-invasive and label free vibrational spectroscopy techniques—including Fourier Transform Infrared (FTIR) and Raman microscopy—stand out.

Vibrational spectroscopy exploits the interaction between light and molecules to probe their vibrational modes in order to obtain a “chemical fingerprint” of a sample. Both FTIR and Raman have been used to monitor modifications to or changes in expression of specific biomolecules, such as DNA levels during cell cycle (Boydston-White et al., 1999; Holman et al., 2003; Short et al., 2005; Swain et al., 2008; Morrish et al., 2019), protein modifications (Chen et al., 2008; Zhang et al., 2015) and DNA damage (Meade et al., 2010; Lipiec et al., 2013, 2014a, b). However, it is becoming apparent that, although it is possible to identify specific signals associated with intracellular biochemical changes, a whole range of subtle spectral variations characterize cell state changes. This is not surprising, as cellular responses induce a swarm of transcriptional up- and down-regulation orchestrating changes to the transcriptomic and proteomic profile of the cell. Using multivariate analysis, spectral information enables classification of cell states or phenotypes of mammalian (Wood et al., 2000a, b; Brown et al., 2009; Smith et al., 2010; Zoladek et al., 2010; Pully et al., 2011; Ramoji et al., 2012; Mazur et al., 2013; Maguire et al., 2015; Hobro et al., 2016; Ichimura et al., 2016; Pavillon et al., 2018), bacterial, and yeast cells (Germond et al., 2018; Kobayashi-Kirschvink et al., 2018).

It is this label-free classification that has great potential both in (i) clinical settings, for disease diagnosis and prognosis, and (ii) in biomedical research, for example in cell sorting for downstream processes. How powerful this tool can be, depends upon our understanding of the correlation between the spectral output and the underlying biochemical pathways within the cells. In bacterial research, antibiotic resistance is of great interest. Spectral markers of antibiotic resistance have been identified at the population level (Germond et al., 2018), and more recently a correlation between peak intensities and expression levels of antibiotic resistance contributing genes has been found (Germond et al., 2018). Importantly, this was done in the absence of antibiotics, indicating that the transcriptional profile of the given cells, affected on its turn by environmental changes (Smith et al., 2018), rather than their phenotypic response to the presence of antibiotics, were responsible for the spectral signatures (Germond et al., 2018). This correlation between Raman spectra and transcriptomic data has further been explored in a comprehensive manner in yeast where it has been shown that Raman spectra and transcriptomic data are linearly correlated (Kobayashi-Kirschvink et al., 2018). In both yeast and bacterial cells, several environmental conditions have been examined. A linear transformation matrix describing the relationship between the Raman data and the transcriptomic data has made it possible to predict an environment-specific Raman spectrum based on transcriptomic data. Conversely, the transcriptome of specific environment has been predicted based on the Raman data (Kobayashi-Kirschvink et al., 2018).

Transcriptomic readout consists of thousands of RNA transcripts, whereas Raman spectroscopy can measure the phenotypic expression of the RNA transcripts, i.e., the biochemical result of the transcriptomic profile. Noteworthy, all transcripts do not change independently; instead, strong correlations are found between transcripts that are controlled by global regulators, reflected in the Raman signals. In the yeast study, it has been determined that only 17 transcripts are sufficient for determining a linear correlation with the Raman spectra. The transcripts largely responsible for the linear correspondence have been identified by determining the variable importance in projection (VIP) values for each transcript. The top scoring transcripts were primarily non-coding RNAs in yeast and ribosome-related transcripts in bacteria (Kobayashi-Kirschvink et al., 2018). The correlation between them does not mean that the Raman spectra directly measure the expression levels of the transcripts in question. Instead, the downstream effects—i.e., changes to expression levels of large groups of genes and the resulting change in biochemical composition of the cells—are quantified by Raman. Thus, by analyzing the correlation between Raman spectra and transcript expression levels, the key cellular pathways affecting the biochemical profile assessed by Raman spectroscopy may be identified.

To our knowledge (i) the correlation between Raman spectra and transcriptomic readouts has never been studied in mammalian cells, and (ii) it has not been examined in the context of cell differentiation. To explore this correlation in mammalian cells, B lymphocytes were chosen as a model cell system. Immune activation of these cells initiates large-scale changes to the transcriptomes, resulting in the differentiation of naïve B cells into mature B cells and class switch recombination (CSR) of the immunoglobulin receptor. Furthermore, as CSR requires reorganization of the DNA, it is a highly regulated process. A large number of regulatory proteins and RNAs have been shown to be involved (Wang et al., 2006; Vigorito et al., 2007; Stanlie et al., 2010; Xu et al., 2010; Zheng et al., 2015; Ramachandran et al., 2016; Sheppard et al., 2018). However, the complex coordination of regulatory pathways and expression modulations is not yet fully understood. Novel techniques and approaches are needed to identify key regulatory RNAs and proteins previously unlinked to the B cell activation differentiation process.

## Materials and Methods

### CH12F3 Cell Culture and Immune Activation

CH12F3 cells were cultured in RPMI 1640 medium with 10% fetal bovine serum, 5% NCTC-109, 1% Pen-Strep, 1% glutamine, 1% sodium pyruvate and 50 μM β-mercaptoethanol. The cells were immune activated by incubating them with a cytokine cocktail (CIT) consisting of 2.5 μg/ml anti-CD40, 10 ng/ml IL-4, and 50 ng/ml TGFβ.

### Flow Cytometry and Antibodies

Flow cytometry measurements, for monitoring class switching assays of CH12F3 cells, were performed using a BD Accuri C6 Plus flow cytometer. 1% PFA fixed CH12F3 cells were stained with FITC Anti-mouse IgA antibody and APC Anti-mouse IgM antibody, both 1:200 dilution.

### Microfluidic Device Preparation

A microfluidic silicon mold was designed to create a small reservoir chip for maintaining cell viability during Raman measurements as previously described (Pagliara et al., 2011). This mold was then replicated in polydimethylsiloxane (PDMS) using a 9:1 ratio of base-to-curing agent. The PDMS was heated at 70°C for one hour and cut to size. A 1.5 mm biopsy punch was used to create an inlet and outlet within the reservoir. The chip was bonded to a glass coverslip using surface ionization by oxygen plasma treatment (10 s exposure to 30 W plasma power in 1 mbar of air).

### Sample Preparation and Raman Mapping

Cells were washed in PBS, pelleted, and resuspended in PBS. PBS was flowed into the bonded chip using Portex tubing PE 0.86 × 0.33mm BxW using a syringe and a 21G microlance. Cells were flowed into the chip in the same way and left to settle for minimum 30 min. Raman maps were collected using a WITec alpha300R confocal Raman microscope system consisting of a 532 nm laser at 16 mW, a fiber-coupled UHTS spectrometer and an optical microscope with a 0.7 NA, 50 × objective. The microfluidic chip containing the cells in PBS suspension was held coverslip side facing up in a custom holder. Single cells (adhering to the glass) were identified in white light imaging mode, then the focus was adjusted in Raman mode using the oscilloscope to maximize the scattered signal intensity (of the C-H stretching peak in the range 2,700–3,000 cm^–1^), and Raman maps were acquired with a 200 nm step size (5 measurement points per micrometer) using a 0.1 s integration time per point. Cells were kept in the microfluidic chip for a maximum of four hours during measurements, before a new chip with fresh cells was prepared.

### Raman Data Analysis

Data processing was performed using MATLAB 2020a. Common k-means analysis with 10 clusters was performed on 118 cell maps (58 × D0, 60 × D4). This involved the calculation of similarity measures between each of the spectra from all 118 maps (a total of 716,250 spectra), each map was either 15 × 15 μm (75 × 75 pixels) or 20 × 20 μm (100 × 100 pixels). As the most similar spectra are grouped together, the spectrum of the new group becomes the mean of its members. At the end of the process, when ten similar group clusters remained, they contained the spectra from regions of cells with similar biochemical constituents, each represented by a mean spectrum or centroid. Each of the 10 clusters was assigned as nucleus, cytoplasm or background, based on the spectral profiles of the cluster centroids. All the spectra that made up the nuclear, cytoplasmic and background spectra were separately averaged for each map. Hence, a mean nucleus, cytoplasm, whole cell (cytoplasm + nucleus) and background spectrum was extracted from each cell map. An array of 13 maps (5 × D0, 8 × D4) was discarded from the dataset before further analysis, since they either contained no pixels identified as nucleus or cytoplasm, or very few pixels associated with nucleus—with a nucleus size of less than 3 μm.

To remove the background signal (from the coverslip and PBS), the background spectra were subtracted from the nucleus and cytoplasm spectra for each map in three steps. (1) The spectra were baseline corrected by subtracting an offset (based on the mean intensity in the range 1,780–1,840 cm^–1^). (2) The background spectra were smoothed using a Savitzky-Golay filter (order = 2, framelength = 99) to reduce the effect of noise. (3) The smoothed background spectra were then subtracted from the nucleus, cytoplasm and whole cell spectra.

Principal Component Analysis (PCA) was performed on nucleus, cytoplasm and whole cell spectra. For each principal component, a *t*-test was used to determine if scores were significantly different between D0 and D4 cells. Linear Discriminant Analysis (LDA) was also performed to calculate a supervised classification model based on a combination of the PC scores. The resulting linear discriminant function could be used to single out the key peaks responsible for the discrimination between D0 and D4 cells.

### RNA Extraction

Total cell RNA was extracted by TRIzol followed with chloroform for phase separation and 100% isopropanol for RNA precipitation. Total RNA was eluted in 30 μl RNase-free water after being washed twice in 75% ethanol. The RNA concentration was assessed using a NanoDrop 2000 spectrophotometer (Thermo Scientific, Waltham, MA, United States). The RNA yield and size distribution were analyzed using an Agilent 2200 Tapestation with RNA Screentape (Agilent Technologies, Foster City, CA, United States).

### RNA-Seq Library Preparation, Next-Generation Sequencing, and Data Processing

For small RNA library preparation, RNA aliquots were used for library preparation using NEBNext Multiplex Small RNA library preparation kit (New England Biolabs, Ipswich, MA, United States). The PCR amplified cDNA construct (from 140 to 160 bp) was purified using a QIAquick PCR Purification kit (Qiagen). The purified cDNA was directly sequenced using an Illumina MiSeq 2000 platform (Illumina, San Diego, CA, United States).

For long RNA library preparation, libraries were constructed using Ribo-Zero Magnetic Gold Kit (Human) (Illumina, San Diego, CA, United States) and NEBNext^®^ Ultra^TM^ RNA Library Prep Kit for Illumina (New England Biolabs) according to the manufacturer’s instructions. Libraries were tested for quality and quantified using qPCR (Kapa Biosystems, Woburn, MA, United States). The resulting libraries were sequenced on a HiSeq 2500 instrument that generated paired-end reads of 100 nucleotides.

Raw sequencing reads were checked for potential sequencing issues and contaminants using FastQC. Adapter sequences, primers, number of fuzzy bases (Ns), and reads with quality scores below 30 were trimmed. Reads with a length of less than 20 bp after trimming were discarded. Clean reads were aligned to the mouse reference genome (GRCm38, 53,465 annotated genes in total) using the TopHat 2.0 program, and the resulting alignment files were reconstructed with Cufflinks (Trapnell et al., 2012). The transcriptome of each sample was assembled separately using Cufflinks 2.0 program.

### Sequencing Data Analyses and Statistical Methods

Read counts of each sample were subjected to cluster analysis (Metsalu and Vilo, 2015) and differential expression analysis using RNA-seq 2G (Zhang et al., 2017). Genes with | fold-change| ≥ 1, *P*-value ≤ 0.05 and false discovery rate (FDR) ≤ 0.05 were considered statistically significant. Expression of significant differentially expressed genes in different B cell subsets was determined using My Geneset ImmGen (Heng et al., 2008). Interaction and gene targets of identified DE ncRNAs in cells and paired EVs were predicted by miRNet and ENCORI (Li et al., 2014; Fan et al., 2016). ncRNAs-target interaction network was constructed by Cytoscape v3.8.0 (Shannon et al., 2003).

### Partial Least Squares Regression

To analyze the potential correlation between the Raman data and the transcriptomic data, Partial Least Squares (PLS) regression was applied to the datasets. The transcriptomic data consisted of the read counts for 17,725 transcripts with three D0 samples (D0-1, D0-2, and D0-3) and three D4 samples (D4-1, D4-2, and D4-3). Dimension reduced Raman data were used in the form of PC scores. To correspond to the three replicates for each condition of the transcriptomic data, the Raman cell measurements were randomly assigned to three groups of D0 and three groups of D4.

PLS regression analysis was performed with a leave-one-out approach; each of the six samples was removed in turn. Each leave-one-out analysis determined a linear regression model between the Raman (R_–*i*_) and transcriptomic (T_–*i*_) datasets—meaning the PLS regression coefficients matrix, β_−*i*_ was found, so that

$$R_{-i}=\beta_{-i}\cdot T_{-i}$$

For each PLS regression analysis, Raman PC scores were predicted for the left-out sample, i, using the transcriptomic data (T_*i*_) and the β_*–i*_ matrix.

To assess the validity of the predicted Raman PC scores (and thus the regression model), they were compared against the single cell PCA scores. For further assessment, the predicted Raman PC scores were then converted to LDA scores and again compared with the single cell data.

## Results

### Identifying Nucleus and Cytoplasm in Single Cell Raman Maps Using Common k-Means

**FIGURE 1:**
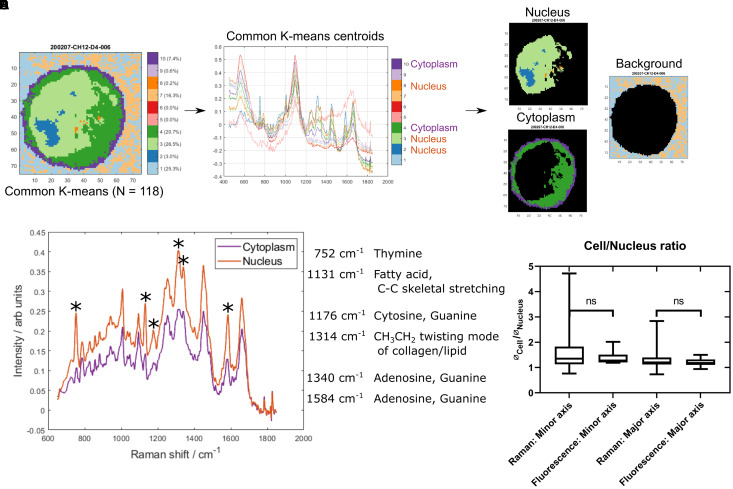
Identifying nucleus and cytoplasm associated areas using common K-means. **(A)** An example cell map (specifically sample 200207-CH12-D4-006) after common K-means with 10 clusters, which was used to analyze 118 individual cell maps concurrently. **(B)** The 10 common K-means centroid spectra. Spectral assignments were used to identify the clusters associated with cytoplasm (4 and 10) and nucleus (2, 3, and 8). **(C)** Example cell map (as seen in **A**) with nucleus (top left), cytoplasm (bottom left), and background (right) associated pixels highlighted. **(D)** Comparison of the mean cytoplasm and nucleus spectrum across all cells. The largest peak differences are highlighted and peak assignments are listed. **(E)** Comparison of nucleus/cell ratio between Raman maps and epifluorescence microscopy images. ^∗^denotes differential peak signals.

Raman maps were collected from 118 live CH12F3 cells suspended in isotonic PBS-filled microfluidic chambers. The cells remained in place throughout measurements. Common k-means analysis was applied to identify cell (vs. background) pixels, as well as to distinguish the nucleus from the cytoplasm within each cell (Figures 1A–C). Additional examples are shown in Supplementary Figures S1A–C. Inspection of the cluster centroid spectra (Figure 1B) informed the segmentation.

The quality of the segmentation was assessed in two ways. Firstly, the mean nucleus spectrum was compared to the mean cytoplasm spectrum (Figure 1D). The most pronounced differences were associated with nucleic acid and lipid/fatty acid signals, with higher intensities found in the nucleus. Secondly, the size of the nucleus relative to the whole cell was assessed and compared with that from epifluorescence microscopy images of CH12F3 cells incubated with nucleic acid stains (Figure 1E). No statistically significant difference was found between the Raman and epifluorescence data. A larger variance was seen for the Raman data—possibly attributed to a number of smaller and kidney shaped nuclei (Supplementary Figures S1F,G). These were not excluded as they were not outliers in the Raman spectral dataset.

### Quantifiable Spectral Differences Between Non-activated and Activated B Cells

**FIGURE 2:**
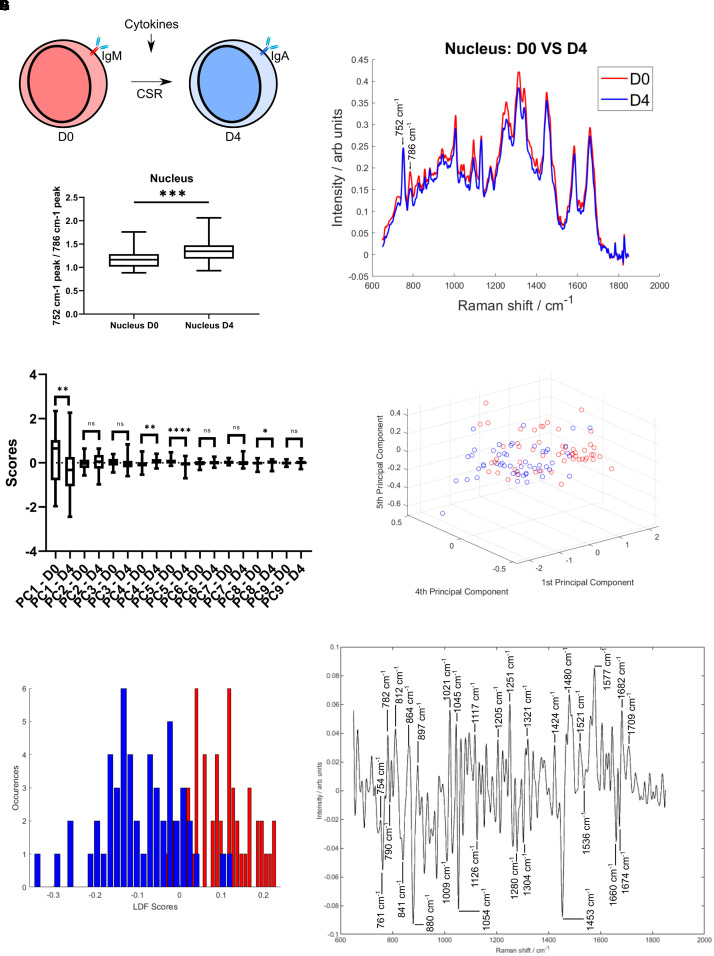
Quantifiable spectral differences between D0 and D4 cells. **(A)** Schematic representation of a CH12F3 cell undergoing class switch recombination in response to exposure to the cytokine cocktail. The expressed B cell receptor constant region changes from IgM to IgA. **(B)** The mean nucleus spectrum of D0 and D4 cells. Two neighboring peaks are highlighted. **(C)** Peak ratio (752/786 cm^–1^ peak). A *t*-test gave a statistically significant difference between samples (^*ns*^*P* > 0.05, ^∗^*P* < 0.05, ^∗∗^*P* < 0.01, ^∗∗∗^*P* < 0.001, ^****^P < 0.0001). **(D)** Principal Component analysis. Comparison of the first 9 PC scores. A *t*-test was applied to identify the principal components with a statistically significant difference between D0 and D4. **(E)** PCA analysis. Scores plotted for components 1, 4, and 5. **(F)** LDA analysis. Histogram of the distribution in the training model. **(G)** LDA analysis. Loading spectrum.

To assess large-scale chromatin conformational and transcriptomic alterations, two groups of CH12F3 cells were compared: non-activated cells (D0) and cells at 96 h post immune activation with cytokine (CIT: anti-CD40, IL-4, and TGFβ) cocktail (D4), as shown in Figure 2A. The immune activation of the cells was verified by quantifying the percentage of IgM-producing cells vs. IgA-producing cells using flow cytometry, around 40% of CH12F3 expressed switched from IgM to IgA 4 days after CIT activation (Supplementary Figure S2).

A prerequisite for further analysis and correlation with the transcriptomes was the ability to separate D0 and D4 cells based on their Raman spectra. Given the primary focus of the study was the nucleus Raman spectrum, we observed a number of spectral differences are apparent between D0 and D4 cells (Figure 2B). Namely, a peak at 786 cm^–1^ shows a large variation between the two activation states. The neighboring peak at 752 cm^–1^ does not show this variation. Both of these peaks are associated with nucleic acids (Ridoux et al., 1988; Stone et al., 2004; Kendall et al., 2010). The distribution of the 752–786 cm^–1^ peak ratios was found to be significantly different between D0 and D4 cells (Figure 2C). This nucleic acid peak ratio therefore has the potential to provide a measure of activation status through the measurement of changes to DNA within the cells.

To further explore the spectral differences between D0 and D4 cells, multivariate approaches were applied. An unsupervised method, Principal Component Analysis (PCA), showed a separation between D0 and D4 cells (Figures 2D,E and Supplementary Figures S3A–C). Four PCs had a statistically significant difference between D0 and D4 scores. The loading spectra of those showed a range of peaks associated with both nucleic acids, lipids and proteins (Supplementary Figures S3D–G). Nucleic acid peaks around 786 cm^–1^ (PC1) and 752 cm^–1^ (PC1, PC4, and PC5) were amongst these, supporting the use of that peak ratio to distinguish between D0 and D4. Although a number of other nucleic acid peaks were identified, it is clear that intracellular changes of protein and lipid are also drivers for the spectral differences.

A supervised method, Linear Discriminant Analysis (LDA) was then applied, building on the PC scores and determining a classifier to discriminate between D0 and D4 cells. The two groups showed a very good separation (Figure 2F). Using a leave-one-out analysis, it was determined that the LDA classifier had a sensitivity of 73.1% and a specificity of 81.1% for identification of D4 cells. The loading plot for the classifier, representing the spectral data separating D0 and D4 cells, consisted of a range of peaks (Figure 2G). Nucleic acid peaks in the 751–790 cm^–1^ range are again present. The largest peaks include nucleic acid, protein, sugar and lipid, such as guanine and cytosine (782, 1,251, 1,577 cm^–1^; Ruiz-Chica et al., 2004; Liu et al., 2008), phosphodiester (812, 897, 1,424 cm^–1^; Ruiz-Chica et al., 2004), tryptophan (754, 761, 880 cm^–1^; Stone et al., 2002; Huang et al., 2003; Cheng et al., 2005; Shetty et al., 2006; Liu et al., 2008), polysaccharide structure and glucose (841, 1,117 cm^–1^; Gniadecka et al., 1997; Stone et al., 2004; Krafft et al., 2005) and CH_2_ deformation (1,304, 1,321 cm^–1^; Stone et al., 2004; Kamemoto et al., 2010). These results show that is possible to distinguish between D0 and D4 cells based on their Raman spectra. Peaks associated with nucleic acids are important for this separation, but alterations to other biomolecules are also detectable.

### Differing Transcriptomic Profiles of Non-activated and Activated B Cells

**FIGURE 3:**
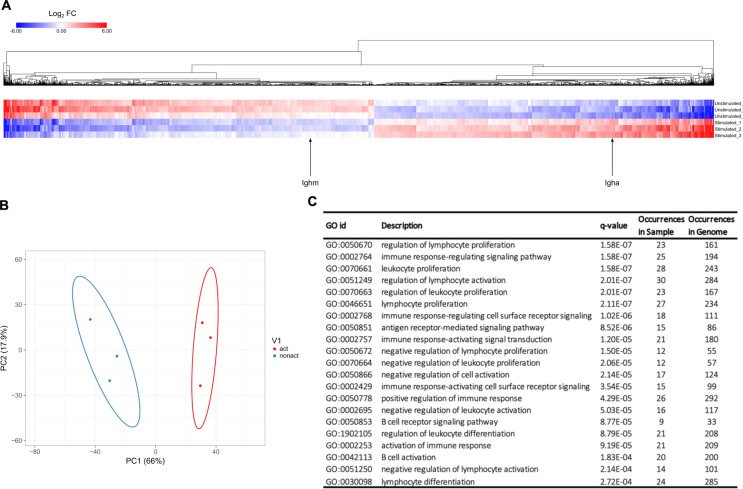
Transcriptomes of D0 and D4 cells. **(A)** Heatmap of the log2 fold change of transcripts from D0 and D4 samples, calculated using the DESeq2 software. Transcripts with | log2 fold change| > 0.5 and FDR < 0.05 are shown. Two transcripts, *Ighm* and *Igha*, are highlighted. *Ighm* and *Igha* code for the immunoglobulin heavy chain constant regions of IgM and IgA receptors, respectively. **(B)** PCA analysis showing separation of D0 and D4 samples. **(C)** Functional enrichment analysis of differentially expressed transcripts between D0 and D4. Table displaying significantly enriched gene ontology (GO) terms and associated biological processes.

The transcriptomic profiles of D0 and D4 cells were determined and analyzed. Read counts were measured for a total of 17,725 transcripts, and differential gene expression analysis using DESeq2 was applied to identify genes that were up- or down-regulated in response to immune activation. Figure 3A shows transcripts with the largest change of expression between D0 and D4 samples clustered based on Euclidean distance from three independent biological repeats which gave rise to expected variance within the three replicates due to difference in cell confluency and number of passages. The two transcripts *Ighm* and *Igha*, which code for the immunoglobulin heavy chain constant regions of IgM and IgA, respectively, are highlighted. *Ighm* expression is down-regulated in D4, while *Igha* is up-regulated. This is a hallmark of the CH12F3 class switching response and in agreement with the IgM to IgA isotype switching measured by flow cytometry (Supplementary Figure S2).

The expression profiles of D0 and D4 cell samples clearly illustrate that several thousands of genes are up- or down-regulated upon immune activation of CH12F3 cells. These data are in line with published results regarding genes that are over-expressed during B cell maturation (Dedeoglu et al., 2004; Huong et al., 2013; Delgado-Benito et al., 2018; Ribeiro de Almeida et al., 2018). For example, we could identify that genes AID, Bcl11a, CD40, and Ccr6 go up 2-, 3-, 2. 6-, 8.7-fold in D4 compared to D0, respectively. This further illustrates the validity of our RNA-seq results. Further whole transcriptomic profiling, including PCA analysis (Figure 3B) and gene ontology analysis (Figure 3C), number of genes from our data occurred in the indicated biological pathways was compared to the number of occurred annotated genes from the whole genome. These datasets demonstrated a clear separation between D0 and D4 samples and the validity of our experimental approach.

PCA analysis on the expression profiles of D0 and D4 CH12F3 cohort samples were performed using Clustvis software tool (Metsalu and Vilo, 2015). Differences were assessed after log_2_ transformation of normalized read counts at a threshold of *p* < 0.05 for multiple comparisons. The variation of expression profile between D0 and D4 CH12F3 was displayed in first and second dimensions (PC1 vs. PC2). Statistical significance was set at false discovery rate (FDR) < 0.05. As a result, we could observe that the three D0 samples cluster in proximity together, as do the three D4 samples. But the D0 vs. D4 clustered markedly separately from each other when plotted on the same graph (Figure 3B). This further validates the distinction in overall transcriptional profile of our cohorts. To further identify the specific basis for this distinction, we used Genemania pathway analysis tool (Warde-Farley et al., 2010). After including all hits in D4 expressed at log_2_ fold change > 1 with FDR < 0.05, we identified the top pathways to include the chemokine signaling pathway, leukocyte activation, immune cell differentiation, and B cell activation amongst other B cell related processes (Figure 3C). This further validates the specificity of our experimental design and its consistency.

### Linear Correlation Between Transcriptomic and Raman Data

Alterations in gene expression can ultimately cause changes in intracellular protein levels, as well as in other biomolecules through changes to metabolic pathways and intracellular structures. All these changes are bound to affect Raman spectral readouts. That a correlation exists between transcriptomic data and Raman spectra, as demonstrated for yeast and bacteria (Kobayashi-Kirschvink et al., 2018), is therefore not unexpected, albeit it was hard to predict whether a linear correlation would exist in a complex mammalian cell such as a B lymphocyte.

**FIGURE 4:**
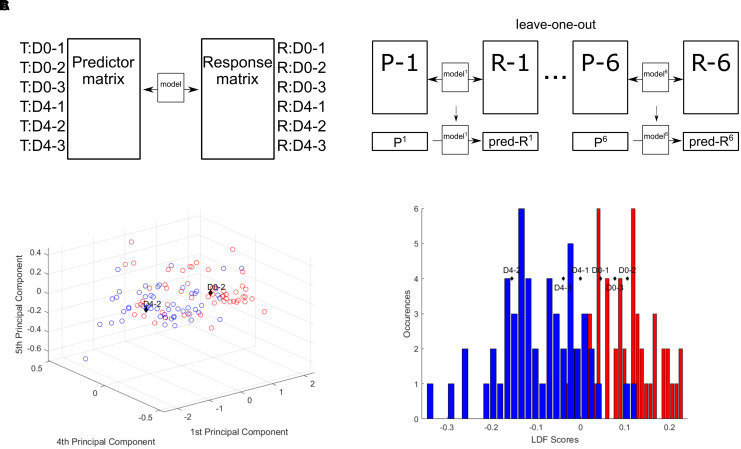
Partial least squares regression model correlates Raman and transcriptomic data. **(A)** Partial least squares regression model. **(B)** Raman scores predicted from transcriptomic read counts. Predicted D0-2 and D4-2 plotted with the single cell scores from PC1, PC4, and PC5. **(C)** Predicted Raman scores converted to LDA scores and plotted against the LDA scores histogram.

To test this hypothesis, a Partial Least Squares (PLS) regression analysis was applied to create a model for the prediction of Raman data from transcriptomic data of CH12F3 cells. A PLS regression model was determined from three D0 samples (D0-1, D0-2, D0-3) and three D4 samples (D4-1, D4-2, D4-3) of transcriptomic and Raman data (Figure 4A and Supplementary Figure S4A). Using a leave-one-out approach, the validity of the linear regression model was tested on each sample in turn; one sample, i, was left out, and a PLS regression coefficients matrix, β_−*i*_, was determined from the remaining five samples. This matrix was then used to predict the Raman scores of the left-out sample.

$$R_{predicted}=\beta_{-i}\cdot T_{i}$$

To assess the validity of these predictions and thus the PLS regression model, the predicted Raman data were compared to the PC scores for each individual cell from the original PCA of the training cell dataset for each sample. Plotting the predicted PC scores against the single cell scores shows D0-2 and D4-2 within their expected regions (Figure 4B) and the rest around the intersection between D0 and D4 (Supplementary Figures S4B–E). Further, by converting the predicted PC scores into their respective LD scores, the predicted group membership (D0 or D4) could be assessed (Figure 4C). The three D0 samples are found within the D0 region, while the D4 samples are found within the D4 region.

These results show that a linear correlation exists between transcriptomic profiles and Raman spectra of CH12F3 cells. Specifically, the variation in transcript expression levels between D0 and D4 cells is reflected in variation in Raman spectra of D0 and D4 cells—and transcriptomic data can be used to predict Raman data of CH12F3 cells.

### Identification of Key Transcripts for the Correlation Between Raman Data and Transcriptomic Data

**FIGURE 5:**
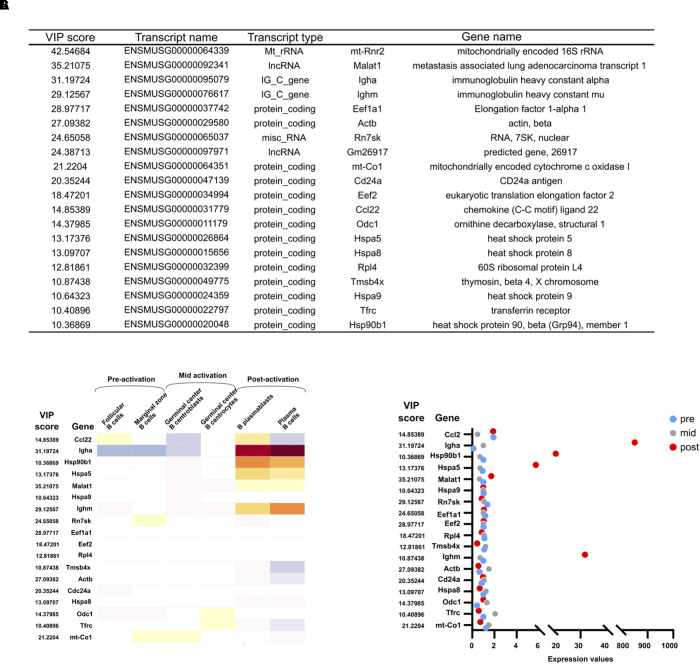
VIP transcript list and their expression *in vivo*. **(A)** The 20 transcripts with the highest Variable Importance in Projection (VIP) scores. **(B)** Expression of VIP genes *in vivo*. Median normalized gene expression values in different B cell subsets *in vivo* as annotated in Immunological Genome Projects. Further subclassified into pre-, mid-, and post-activation correlating to activation for CSR in CH12F3. **(C)** Quantification of normalized expression values of VIP genes in the dot plot.

The importance of each transcript for the regression model is of particular interest, as this may reveal genes or pathways that are essential for the immune activation process. As shown in Figure 3, thousands of transcripts are significantly differentially expressed between D0 and D4. However, translation levels, protein modifications, and other regulatory mechanisms add further complexity to the final biochemical composition of the cell. A transcriptional profile does not account for these additional layers of regulation. Identifying transcripts of high importance for the correlation with the intracellular biochemical changes as measured by Raman microscopy may therefore be of great value. The Variable Importance in Projection (VIP) score was determined for each transcript. The top 20 transcripts for the PLS regression are shown in Figure 5A. We term these hits the VIP list, as it consists of the 20 transcripts with the highest importance for the PLS regression model which enables the prediction of Raman scores from transcriptomic read counts. Upon further analyses of the protein coding entries in our VIP list, we could identify that many of our identified hits do indeed correlate with expression profiles from *in vivo* activated B cells isolated from murine germinal center splenocytes (Figure 5B). Moreover, their expression quantifications (Figure 5C) correlate with post-activation B cell responses. It is worth noting that germinal center splenocytes and CH12F3 cells are not directly comparable given the immortalized nature of the CH12F3 cell line. That is why we configured the *in vivo* germinal center B cell response into three broad groups that we termed pre-, mid-, and post-activation (Figures 5B,C). We also took two representative *in vivo* cohorts for each of these three broad groups as represented in Figure 5B to ensure maximum congruency between *ex vivo* and *in vivo* analyses.

*Ighm* and *Igha* are both found in the top four gene hits of the VIP list (Figure 5A). Although their change in expression levels results in the isotype switching from IgM to IgA, it is worth noting that they by no means are the most differentially expressed genes (Figure 3). Their high importance for the correlation with the Raman data therefore underlines that the transcripts with the highest fold change are not necessarily the most informative of the changing biochemical composition of a cell. The importance of IgM and IgA expression in the immune activation process is obvious, and their high presence on the VIP list supports the validity of the PLS regression model. Additional transcripts in the top 20 include regulatory and ribosomal RNAs, which is in line with data from yeast and bacterial analysis (Kobayashi-Kirschvink et al., 2018). A large number of regulatory proteins are also on the list, including a number of heat shock proteins, which have previously been shown to be important for CSR (Orthwein et al., 2010). Actin is also high on the list, in agreement with studies showing a regulatory role of the actin cytoskeleton in B cell activation (Song et al., 2014; Li et al., 2019), as well as possibly the role of monomeric actin in DNA damage response (DDR) and chromatin modifications which happens during cell development or DNA repair (Baarlink et al., 2017; Caridi et al., 2019; Hurst et al., 2019).

A PLS regression model was also determined for whole cell and cytoplasm Raman data (Supplementary Figures S5A,B). These allowed for predictions in line with the nucleus data (cytoplasm shown in Supplementary Figures S5C–H). The top 20 VIP transcripts for those models (Supplementary Tables S1, S2) were largely identical to the nucleus list. The values and order of the transcripts varied slightly, but only one transcript differed between the nucleus and whole cell list. For cytoplasm compared to nucleus, only three transcripts were different in the top 20 hits.

## Discussion

The cell segmentation used to isolate nucleus and cytoplasm regions within each cell using the Raman spectral maps proved useful for highlighting DNA Raman peaks. The 752/786 cm^–1^ peak ratio, shown to be statistically different between D0 and D4, has potential as a measure of activation status. If it is to be used as such, the biological significance of this peak ratio is of interest. The structure of DNA likely plays a role here. There are three biologically relevant double helical structures of DNA: A-DNA, B-DNA, and Z-DNA. B-DNA is the most common. A Raman peak at ∼784–787 cm^–1^ has been shown to have a strong intensity for B-DNA, but much lower for the other two. The peak consists of two subpeaks; the breathing mode of the cytosine ring and the phosphodiester symmetric stretch of B-DNA backbone (Benevides and Thomas, 1983; Ridoux et al., 1988). During B to Z transition of DNA, the phosphodiester symmetric stretch signal downshifts (Ridoux et al., 1988). As Z-DNA is associated with the rate of transcription (Wittig et al., 1991; Shin et al., 2016), it is plausible that the restructuring of DNA during activation could account for the difference between D0 and D4.

From the analysis of the whole Raman spectrum and associated trend of change in nuclear contributions, it is possible that the spectra identified as from the nucleus are unlikely to be pure spectra, completely free of cytoplasmic signal. The similar VIP transcript lists of especially “nucleus” and “whole cell” analysis support this. Although a confocal microscope was used, optical signal from cytoplasm above and below the nucleus was likely measured too. The relatively large nucleus in CH12F3 cells and the round shape of the cells could have contributed to this. For larger and flatter adherent cells with smaller nuclei relative to the overall cell size, this may not occur to the same extent. Here it could be interesting to determine if a PLS regression model and its top VIP transcripts differed more between distinct cellular regions than for CH12F3 cells.

Both the PCA and LDA analysis revealed a myriad of spectral differences that allowed for the classification of D0 vs. D4 cells. These included a large number of nucleic acid associated peaks, but also protein, lipid and sugar peaks. Classification of cell types or cell states based on Raman spectra has great clinical and research potential. However, understanding the biological significance of the spectral changes is of importance if these tools are to be implemented as a standard technique in biological laboratories. Peak assignments based on single molecule measurements provide some help with interpretation of the spectral changes. Correlation with transcriptomic data and identification of top VIP transcripts could add further value to the Raman data.

Here we showed that a linear correlation between Raman data and conventional next generation transcriptomic data exists in CH12F3 cells. Raman data were predicted based on transcriptomic profiles. When comparing the predicted Raman data to single cell data, the classification of each prediction was within the expected groups (D0 vs. D4). We also identified the transcripts with the highest importance for the correlation with Raman spectra of non-activated and activated CH12F3 cells (Figure 5A). The immunoglobulin genes *Ighm* and *Igha* both featured in our top hits, highlighting the value of the PLS regression model as a valid phenotypic measurement for B cell activation. A number of regulatory RNAs and proteins were also in the top 20, some known to be involved in the regulation of CSR and activation, and others not previously shown to be involved. Further experiments exploring the role of these transcripts in B cell activation and CSR could be of great interest in the field of adaptive humoral immunity. This also suggests that our methodology could have the potential to identify novel molecular factors that other conventional assays might miss. One possible reason is the ability of our assay to combine both qualitative and quantitative analysis of nuclear signals along with a phenotypic readout of the overall status of the nucleus at the single cell level. RNA-seq, on the other hand, primarily measures quantitative readouts of bulk cells. Supported by similar results previously achieved in yeast cells (Kobayashi-Kirschvink et al., 2018), our work could provide a compelling argument for the use of Raman microscopy for phenotypic screening of a range of complex cellular processes. Additional time points between D0 and D4 could further elucidate the correlation between Raman spectra and transcriptomic profiles. As there is some, although minor, inter-sample variability due to confluency levels and number of cell passages, it may also be beneficial to extract RNA and measure Raman spectra of cells from the same population on the same day.

Moving forward, it would be worth correlating Raman data to transcriptomic data in a reverse order to increase confidence of the data validity, but also to potentially provide a rapid, non-destructive, surrogate measure of potential transcriptomic profile from a known cell type. It would also be very interesting in the future to combine single-cell RNA-seq with Raman measurements to provide a more specific and accurate correlation. Especially, allowing us the potential to predict transcriptomic profiles from Raman imaging data, which would be an extremely powerful future Raman application tool. To accomplish this though, it might be important first to: (i) compare sufficient numbers of single-cell Raman datasets to a commensurate size of single-cell RNA-seq profiles, and (ii) isolate the nuclear vs. cytoplasmic signals to purity, through biochemical isolation of individual cellular nuclei via hypertonic solution cytoplasmic lysis. Ultimately, from the identification of differential Raman spectra differences and signatures, it could be possible to deduce correlating changes in gene expression, such as overall RNA sizes distribution and subtypes (i.e., miRNA, lncRNA, mRNA), and ultimately to predict expression of specific genes. This would be a very powerful approach to unraveling the direct relationship between the two complementary data types. Indeed, there is still much to be determined, but there most certainly is a correlation between transcriptomic profiles and Raman spectra and this correlation can be further enhanced by combining more refined biological techniques. Understanding the origin of this correlation in different case studies will add value to Raman measurements of biological samples and aid interpretation of spectral changes. Furthermore, the work outlined here, suggests that Raman may also aid in the identification of key regulatory transcripts for immune activation. Future work will demonstrate if this can be translated to elucidating other cellular developmental processes, occurring in healthy cells or during disease.

## Data Availability Statement

The data presented in the study are deposited in FigShare, https://doi.org/10.6084/m9.figshare.14135219.v1.

## Author Contributions

FP, RC, SP, and NS conceived and designed the experiments. RM and SP constructed the microfluidic chip. RM performed the experiments and wrote the manuscript with inputs from the other authors. KY acquired the transcriptomic data. RM, KY, and NS analyzed the data. All authors contributed to the article and approved the submitted version.

## Conflict of Interest

The authors declare that the research was conducted in the absence of any commercial or financial relationships that could be construed as a potential conflict of interest.
