# Supplementary material for: Single Cell Label-Free Probing of Chromatin Dynamics During B Lymphocyte Maturation
Source: Front Cell Dev Biol. 2021 Mar 26;9:646616. doi: 10.3389/fcell.2021.646616 (PMC8033168; doi:10.3389/fcell.2021.646616)
Supplement: Supplementary file 1 [file Table_1.DOCX]

**Single cell label-free probing of chromatin dynamics during B lymphocyte maturation**

Rikke Morrish^1,2^, Kevin Ho Wai Yim^3^, Stefano Pagliara^2^*, Francesca Palombo^1^*, Richard Chahwan^3^*, and Nicholas Stone^1^*

*^1^School of Physics and Astronomy, University of Exeter, Exeter, EX4 4QL, United Kingdom*

*^2^Living Systems Institute and School of Biosciences, University of Exeter, Exeter, United Kingdom*

*^3^Institute of Experimental Immunology, University of Zurich, Zurich, Switzerland*

* Equally contributing authors and to whom correspondences should be addressed:

[*s.pagliara@exeter.ac.uk*](mailto:s.pagliara@exeter.ac.uk)*,* [*f.palombo@exeter.ac.uk*](mailto:f.palombo@exeter.ac.uk)*,* [*chahwan@immunology.uzh.ch*](mailto:chahwan@immunology.uzh.ch)*,* [*n.stone@exeter.ac.uk*](mailto:n.stone@exeter.ac.uk)

*Assessing the cell and nucleus segmentation*

The additional cell map examples (Figure S1a-c) provide a clearer idea of how the cell and nucleus segmentation worked. Three clusters were determined to be associated with nucleus. The primary nucleus cluster varies between cell maps. That is also the case for the cytoplasm and background clusters. The Hierarchical Clustering Analysis (HCA) plot (Figure S1d) depicts the relationship between the ten clusters. All three nucleus clusters (2, 3, and 8) are similar to each other. One cytoplasm cluster (4) resembles the nucleus clusters, while the other one (10) is more similar to the background clusters. The ten clusters were assigned to either nucleus, cytoplasm or background based on the centroid spectra (Figure 1b), the HCA plot, and their effect on the cell and nucleus segmentation within each cell map. The cell versus background segmentation was straightforward, as seen in both the example maps and the centroid spectra. Nucleus versus cytoplasm segmentation was based on peaks associated with nucleic acid, which is more abundant in the nucleus. Cluster 4 proved to be the most difficult to assign – it was found to be at the interface between nucleus and cytoplasm, as shown by the example maps and HCA plot. The assignment of cluster 4 to cytoplasm was based on the centroid spectrum and the resulting nucleus size distribution of all maps.

The size and the shape of the nuclei vary between maps, as seen in Figure 1c and Figure S1a-c. This variation was quantified and assessed by measuring the major and minor axes of each nucleus and comparing them to those derived from epifluorescence images (Figure S1e-g). For both major and minor axes, the Raman mapped nuclei were smaller than the epifluorescence images nuclei. As both the measurement techniques and experimental setup were different between the two, this was not of great concern. Indeed, the Cell/Nucleus ratio was similar between the Raman maps and epifluorescence images (Figure 1e).


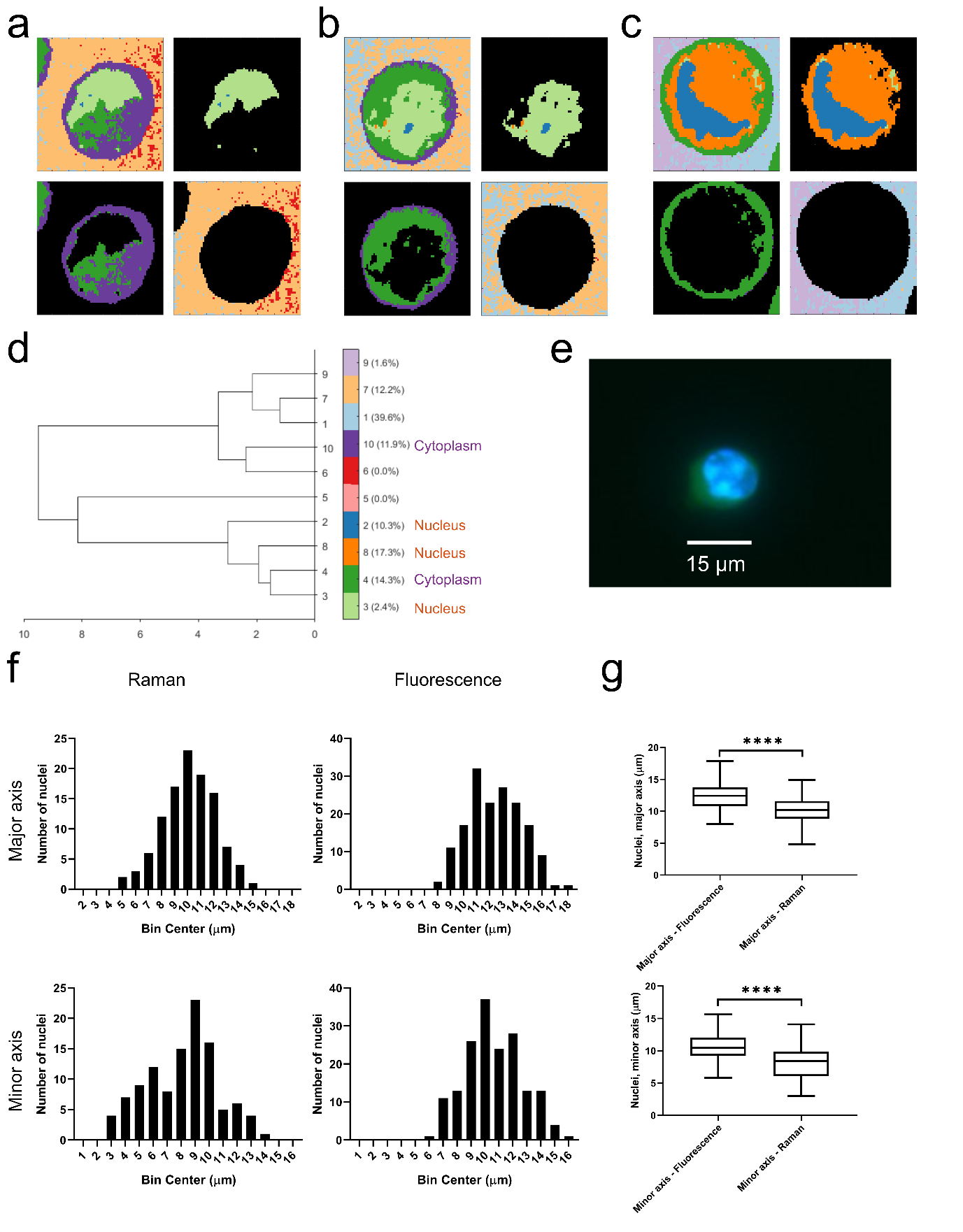


**Figure S1: Assessing the cell and nucleus segmentation**

**(a-c)** Additional example cell maps (specifically samples 200114-CH12-D4-009 (a), 200212-CH12-D0-002 (b), and 200228-CH12-D0-007 (c)) after common K-means with 10 clusters, as presented in Figure 1a-c. Nucleus (top right), cytoplasm (bottom left), and background (bottom right) associated pixels highlighted. **(d)** Hierarchical clustering analysis plot of the common K-means. The clusters identified as cytoplasm and nucleus are annotated. **(e)** Epifluorescence image of a CH12F3 cell: the nucleus is stained with Hoechst (blue) and the whole cell with SYTO13 (green). **(f)** Histograms showing the nucleus size distribution (major axis, left and minor axis, right) of the cells measured from Raman maps (top) and epifluorescence microscopy images (bottom). **(g)** Quantification of (b). A t-test gave a statistically significant difference between samples (ns.: P>0.05, *: <0.05, **: P<0.01, ***:P<0.001).

*Quantifiable spectral differences between non-activated and activated B cells*

CH12F3 CSR in response to CIT treatment was verified by flow cytometry (Figure S2). D0 cells almost exclusively produce IgM BCRs, while a subset of D4 cells have undergone IgM to IgA isotype switching.

PCA analysis was applied to identify the spectral differences between D0 and D4 cells. Four principal components (PC1, PC4, PC5, and PC8) had statistically significant different scores between D0 and D4 cells. Figure S3a-c show the separation between D0 (red) and D4 cells (blue) for these PCs, while Figure S3d-g show their PCA loading spectra.


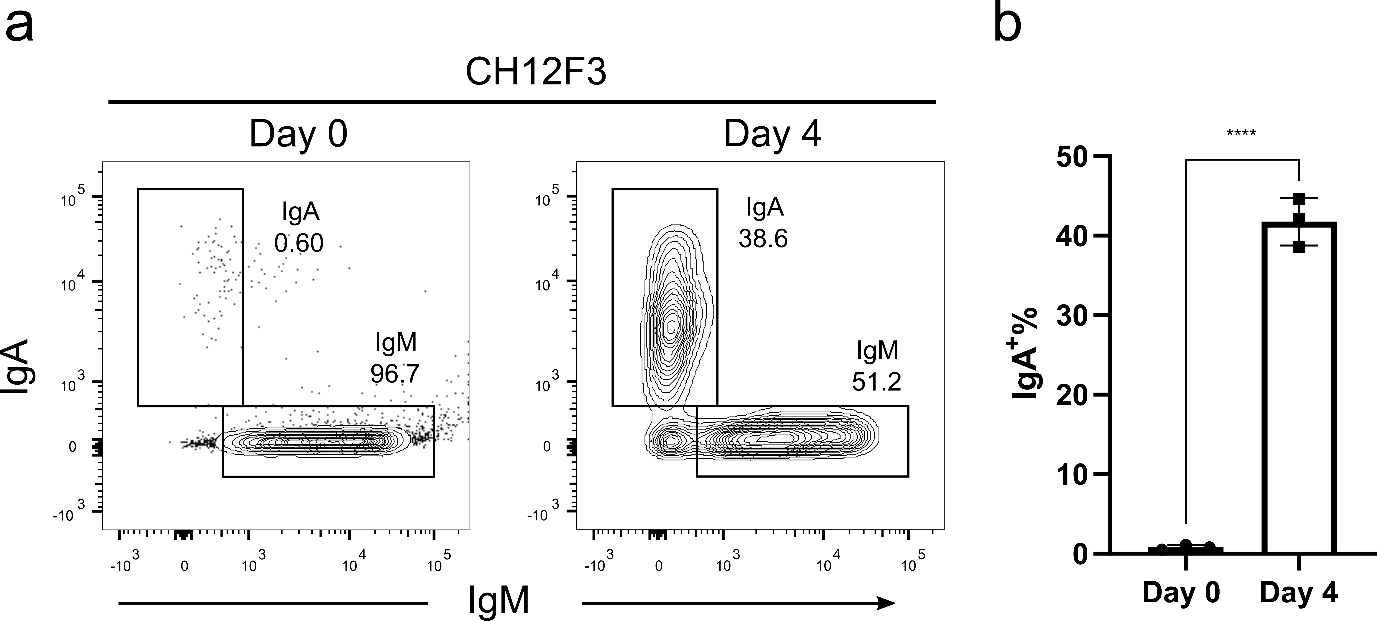


**Figure S2: Monitoring CSR in CH12F3 cells**

**(a)** CSR in CH12F3 upon CIT stimulation monitored by identifying IgM- and IgA-producing cells using flow cytometry. **(b)** Quantification of IgA+ cells for D0 and D4.


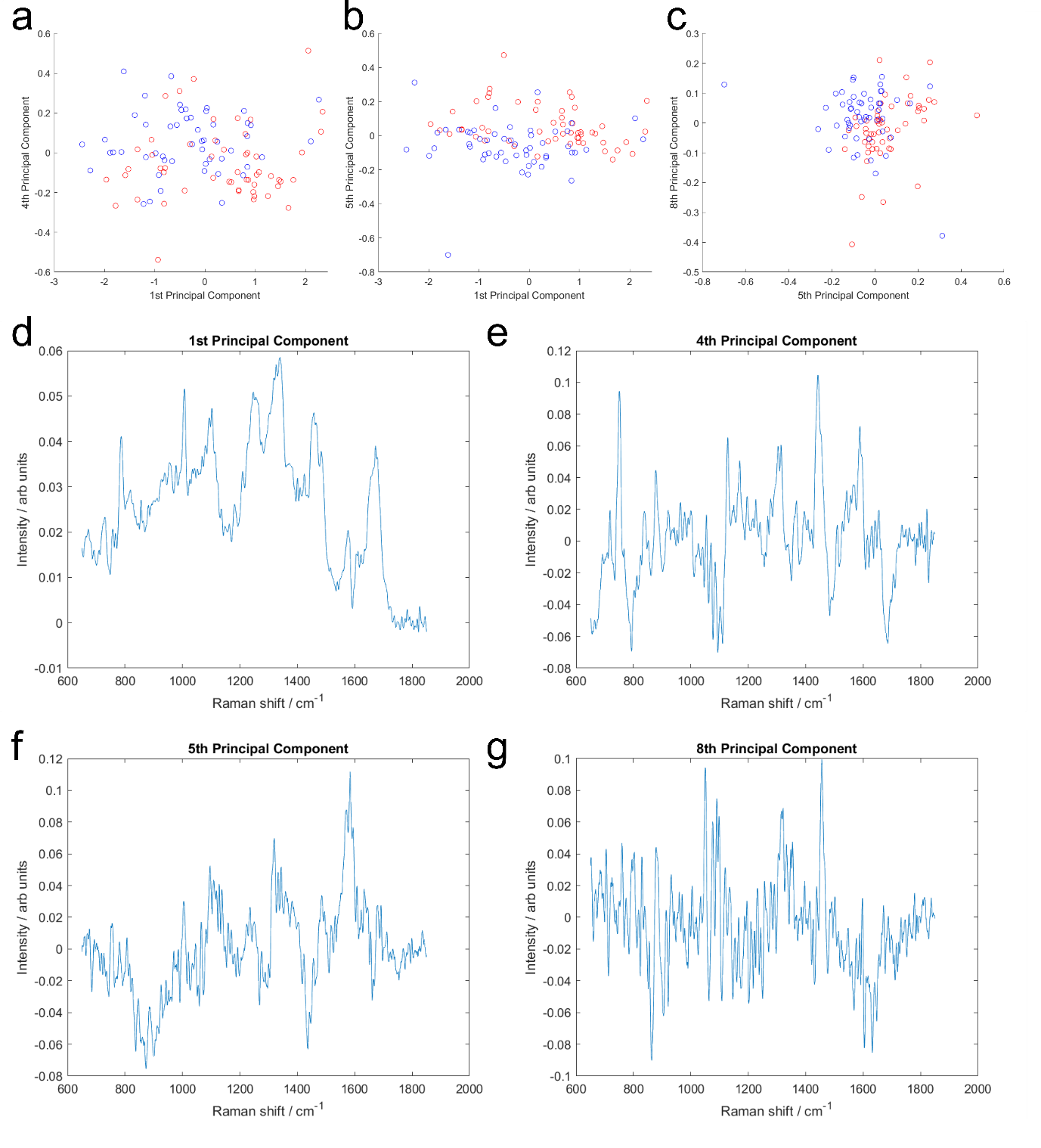


**Figure S3: PCA and LDA analysis for discrimination between D0 and D4 cells**

**(a)** PCA scores; PC1 versus PC4. **(b)** PCA scores; PC1 versus PC5. **(c)** PCA scores; PC5 versus PC8. **(d)** PCA loadings; PC1. **(e)** PCA loadings; PC4. **(f)** PCA loadings; PC5. **(g)** PCA loadings; PC8.

*Predicting Raman data from transcriptomic data using PLS regression models*

The linear correlation between Raman data and transcriptomic data, as determined by the PLS analysis, is visualised in Figure S4a for component 1. The D0 samples cluster together, as do the D4 samples. This is the basis for the model which enabled the prediction of Raman data from transcriptomic data (Figure 4b-c and Figure S4b-e).

In addition to the Raman nucleus data (Figure 4 and Figure S4), Raman whole cell and cytoplasm data were also used for PLS analysis (Figure S5). The whole cell data analysis results were largely identical to those of the nucleus data analysis (Figure S5a). For the cytoplasm data, the results differed more. However, the linear correlation was still clear (Figure S5b), and it was still possible to accurately predict Raman data from transcriptomic data (Figure S5c-f).


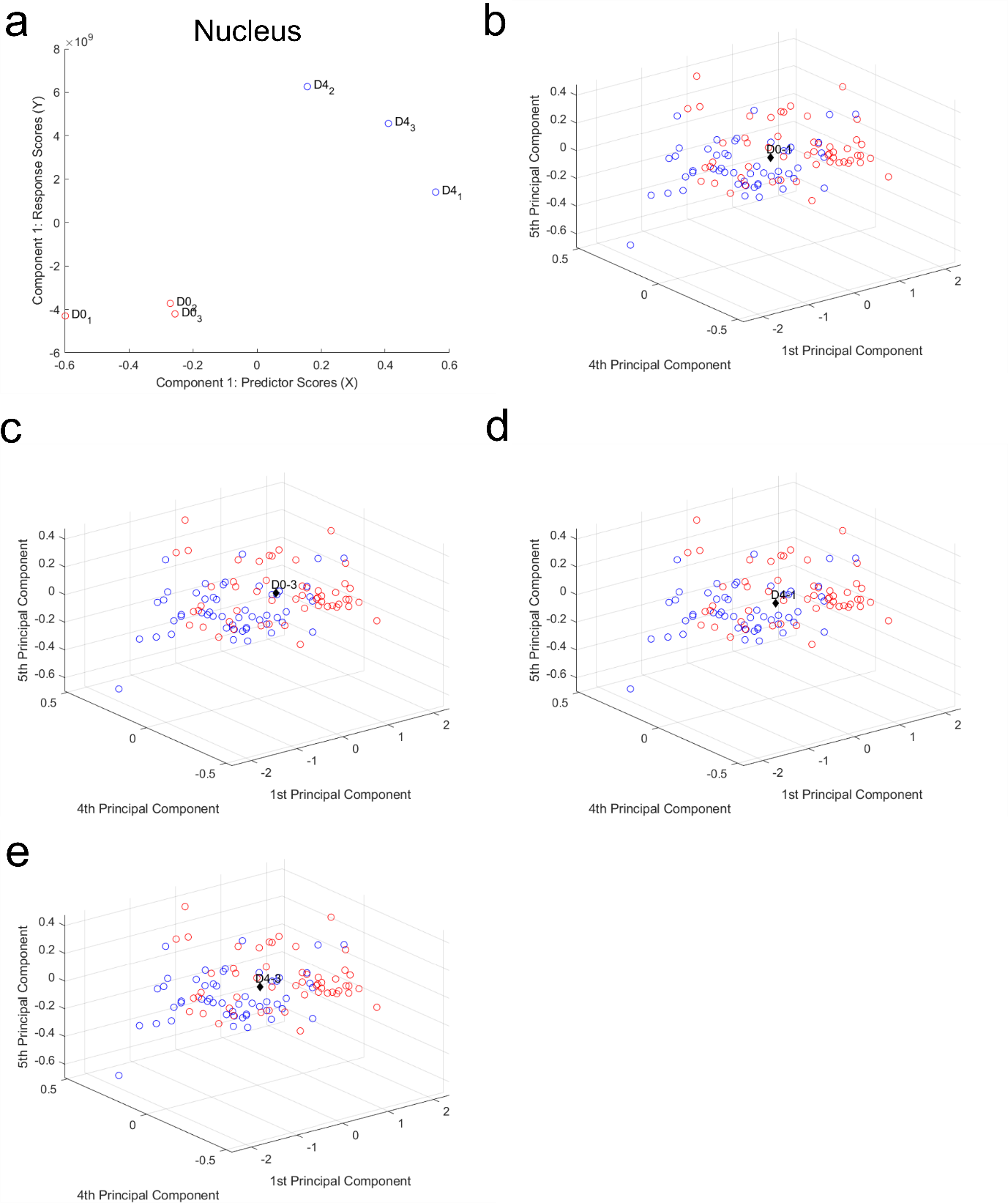


**Figure S4: PLS regression model can predict Raman data from transcriptomic data**

**(a**) PLS regression analysis shows a linear correlation between Raman nucleus data and transcriptomic data for component 1. **(b-e)** Raman scores predicted from transcriptomic read counts. D0-1 (b), D0-3 (c), D4-1 (d), and D4-3 (e) plotted with the single cell scores from components 1, 4 and 5.


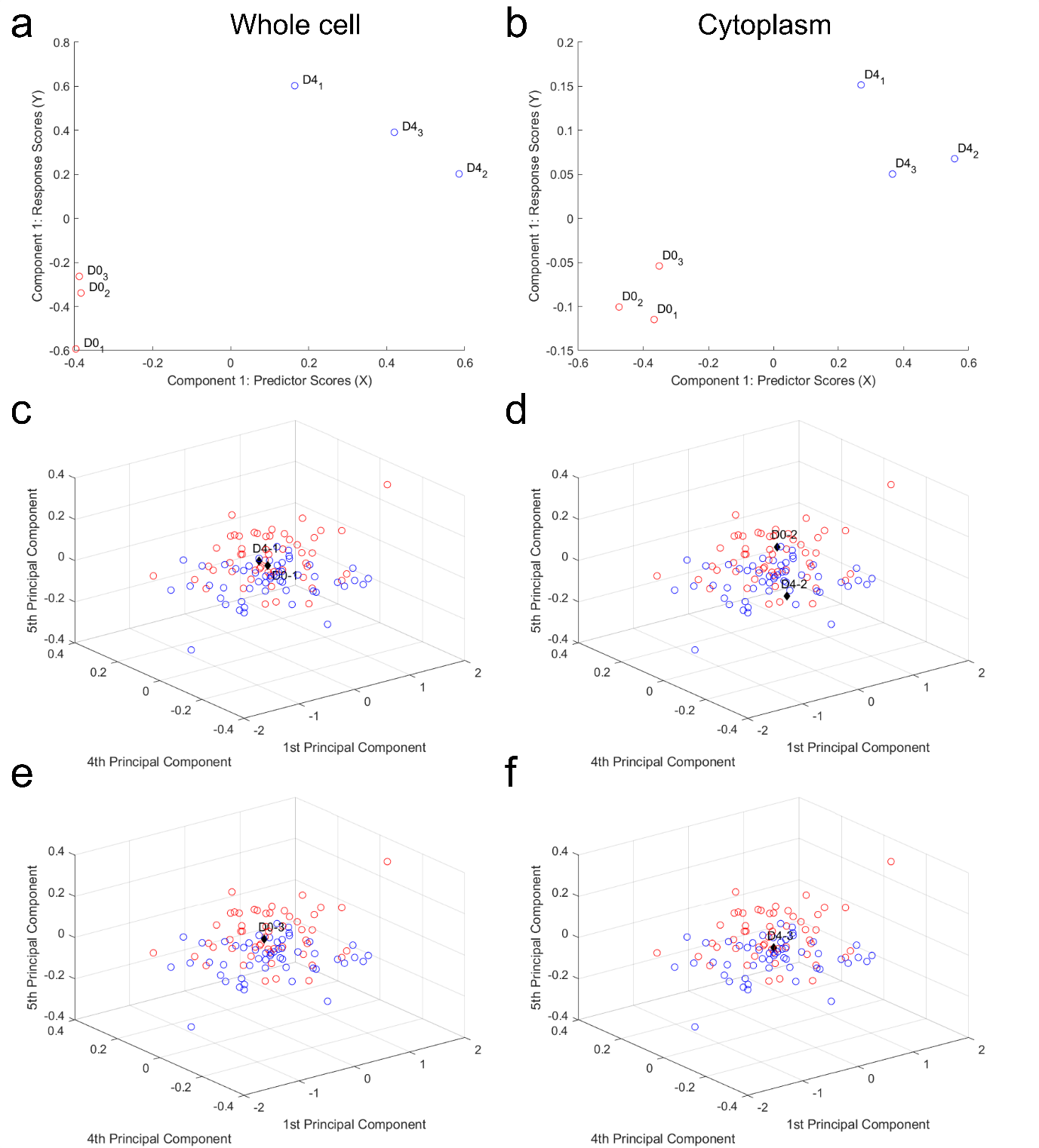


**Figure S5: PLS regression model correlates Raman whole cell and cytoplasm data with transcriptomic data**

**(a)** PLS regression analysis shows a linear correlation between Raman whole cell data and transcriptomic data for component 1. **(b)** PLS regression analysis shows a linear correlation between Raman cytoplasm data and transcriptomic data for component 1. **(c-f)** Raman cytoplasm PLS model: Raman scores predicted from transcriptomic read counts. D0-1 and D4-1 (c), D0-2 and D4-2 (d), D0-3 (e), D4-3 (f) plotted with the single cell scores from PC1, PC4, and PC5.

| **VIP score** | **Transcript name** | **Transcript type** | **Gene name** | |
| --- | --- | --- | --- | --- |
| 41.68549 | ENSMUSG00000064339 | Mt_rRNA | mt-Rnr2 | mitochondrially encoded 16S rRNA |
| 31.63403 | ENSMUSG00000037742 | protein_coding | Eef1a1 | Elongation factor 1-alpha 1 |
| 30.71642 | ENSMUSG00000095079 | IG_C_gene | Igha | immunoglobulin heavy constant alpha |
| 30.42896 | ENSMUSG00000092341 | lncRNA | Malat1 | metastasis associated lung adenocarcinoma transcript 1 |
| 29.63411 | ENSMUSG00000076617 | IG_C_gene | Ighm | immunoglobulin heavy constant mu |
| 28.14179 | ENSMUSG00000029580 | protein_coding | Actb | actin, beta |
| 23.71356 | ENSMUSG00000097971 | lncRNA | Gm26917 | predicted gene, 26917 |
| 23.70888 | ENSMUSG00000065037 | misc_RNA | Rn7sk | RNA, 7SK, nuclear |
| 22.0795 | ENSMUSG00000064351 | protein_coding | mt-Co1 | mitochondrially encoded cytochrome c oxidase I |
| 20.93438 | ENSMUSG00000047139 | protein_coding | Cd24a | CD24a antigen |
| 20.11123 | ENSMUSG00000034994 | protein_coding | Eef2 | eukaryotic translation elongation factor 2 |
| 15.38487 | ENSMUSG00000011179 | protein_coding | Odc1 | ornithine decarboxylase, structural 1 |
| 14.87474 | ENSMUSG00000031779 | protein_coding | Ccl22 | chemokine (C-C motif) ligand 22 |
| 14.05003 | ENSMUSG00000032399 | protein_coding | Rpl4 | 60S ribosomal protein L4 |
| 13.63799 | ENSMUSG00000015656 | protein_coding | Hspa8 | heat shock protein 8 |
| 11.70491 | ENSMUSG00000026864 | protein_coding | Hspa5 | heat shock protein 5 |
| 11.58428 | ENSMUSG00000049775 | protein_coding | Tmsb4x | thymosin, beta 4, X chromosome |
| 11.54382 | ENSMUSG00000024359 | protein_coding | Hspa9 | heat shock protein 9 |
| 10.83353 | ENSMUSG00000057113 | protein_coding | Npm1 | Nucleophosmin |
| 10.81621 | ENSMUSG00000022797 | protein_coding | Tfrc | transferrin receptor |

**Table S1: VIP list for Whole cell Raman data PLS regression analysis**

| **VIP score** | **Transcript name** | **Transcript type** | **Gene name** | |
| --- | --- | --- | --- | --- |
| 36.48182 | ENSMUSG00000037742 | protein_coding | Eef1a1 | Elongation factor 1-alpha 1 |
| 36.38903 | ENSMUSG00000064339 | Mt_rRNA | mt-Rnr2 | mitochondrially encoded 16S rRNA |
| 34.47657 | ENSMUSG00000076617 | IG_C_gene | Ighm | immunoglobulin heavy constant mu |
| 27.54574 | ENSMUSG00000095079 | IG_C_gene | Igha | immunoglobulin heavy constant alpha |
| 27.18289 | ENSMUSG00000097971 | lncRNA | Gm26917 | predicted gene, 26917 |
| 27.03748 | ENSMUSG00000029580 | protein_coding | Actb | actin, beta |
| 24.54971 | ENSMUSG00000065037 | misc_RNA | Rn7sk | RNA, 7SK, nuclear |
| 24.41311 | ENSMUSG00000064351 | protein_coding | mt-Co1 | mitochondrially encoded cytochrome c oxidase I |
| 24.35519 | ENSMUSG00000092341 | lncRNA | Malat1 | metastasis associated lung adenocarcinoma transcript 1 |
| 21.69827 | ENSMUSG00000034994 | protein_coding | Eef2 | eukaryotic translation elongation factor 2 |
| 19.10295 | ENSMUSG00000047139 | protein_coding | Cd24a | CD24a antigen |
| 15.70651 | ENSMUSG00000032399 | protein_coding | Rpl4 | 60S ribosomal protein L4 |
| 15.32205 | ENSMUSG00000011179 | protein_coding | Odc1 | ornithine decarboxylase, structural 1 |
| 13.15234 | ENSMUSG00000031779 | protein_coding | Ccl22 | chemokine (C-C motif) ligand 22 |
| 12.79875 | ENSMUSG00000057113 | protein_coding | Npm1 | Nucleophosmin |
| 12.78879 | ENSMUSG00000015656 | protein_coding | Hspa8 | heat shock protein 8 |
| 12.50920 | ENSMUSG00000058655 | protein_coding | Eif4b | Eukaryotic translation initiation factor 4B |
| 12.18672 | ENSMUSG00000024359 | protein_coding | Hspa9 | heat shock protein 9 |
| 10.85725 | ENSMUSG00000049775 | protein_coding | Tmsb4x | thymosin, beta 4, X chromosome |
| 10.57061 | ENSMUSG00000051506 | protein_coding | Wdfy4 | WD repeat and FYVE domain containing 4 |

**Table S2: VIP list for Cytoplasm Raman data PLS regression analysis**
